# Long-term washover fan accretion on a transgressive barrier island challenges the assumption that paleotempestites represent individual tropical cyclones

**DOI:** 10.1038/s41598-020-76521-4

**Published:** 2020-11-12

**Authors:** Antonio B. Rodriguez, Ethan J. Theuerkauf, Justin T. Ridge, Beth M. VanDusen, Stephen R. Fegley

**Affiliations:** 1 Institute of Marine Sciences, University of North Carolina at Chapel Hill, Morehead City, NC 28557 USA; 2 Department of Geography, Environment, and Spatial Sciences, Michigan State University, East Lansing, MI 48824 USA; 3 Division of Marine Science and Conservation, Nicholas School of the Environment, Duke University Marine Laboratory, Beaufort, NC 28516 USA; 4 School for Environment and Sustainability, University of Michigan, Ann Arbor, MI 48109 USA

**Keywords:** Geomorphology, Sedimentology, Natural hazards

## Abstract

Barrier island overwash occurs when the elevation of wave runup exceeds the dune crest and induces landward transport of sediment across a barrier island and deposition of a washover deposit. Washover deposition is generally attributed to major storms, is important for the maintenance of barrier island resilience to sea-level rise and is used to extend hurricane records beyond historical accounts by reconstructing the frequency and extent of washover deposits preserved in the sedimentary record. Here, we present a high-fidelity 3-year record of washover evolution and overwash at a transgressive barrier island site. During the first year after establishment, washover volume and area increased 1595% and 197%, respectively, from at least monthly overwash. Most of the washover accretion resulted from the site morphology having a low resistance to overwash, as opposed to being directly impacted by major storms. Washover deposits can accrete landward over multi-year time scales in the absence of large storms; therefore, paleotempestites can be more complex than single event beds.

## Introduction

Transport of sediment and water across a barrier island during increased ocean-water levels and wave heights, termed overwash, can be highly detrimental to infrastructure^1^, human health^2^, and economies^3^. Despite those hazards, overwash is essential for sustaining barrier islands faced with rising sea level because it fortifies the island by moving sand landward and depositing it as elevated washover terraces and fans. Washover deposits increase barrier-island width, resilience to sea-level rise, and resistance to erosional events^4^. In addition, washover deposits provide intertidal substrate for saltmarsh, oyster reef, mangrove, and seagrass colonization and supratidal substrate for terrestrial species^5^. Episodic overwash drives barrier island landward migration with sea-level rise maintaining sediment budgets in dynamic equilibrium^6,7^. Persistent erosion narrows and lowers a barrier island across a critical-width and elevation threshold to where storm overwash can deposit washover sediment in the back barrier^8–10^. This storm-induced deposition expands island width and height. Island expansion increases both immediately during overwash events by shifting back-barrier areas into intertidal and supratidal elevations, and gradually through subsequent years by reducing the density of sand-trapping fore-dune vegetation, which promotes aeolian transport of sand landward across the island and the formation of incipient dune fields as grasses reestablish. In the absence of further disturbance, the new sand flats and dunes will provide higher-elevation substrate for vegetation to colonize (saltmarsh and dune grasses) and the vegetation will increase the rate of vertical accretion, decrease aeolian transport of sand across the island and decrease local erosion as roots extend into the subsurface binding sediment^11^.

Overwash is commonly linked with storm conditions, mainly investigated by pairing pre- and post-storm observations^12–16^, during storm observations^17^, and short-term monitoring (< 1 year)^18,19^. The occurrence of non-storm overwash has been documented^20–22^, demonstrating the capacity of overwash to transport sediment across a shoreline in the absence of a major event. The emplacement of a washover deposit in a back-barrier environment (marshes, lagoons, and ponds), however, is generally interpreted as resulting from a single major storm event, such as a hurricane. Washover deposits preserved in the stratigraphic record (paleotempestites) are used to reconstruct the spatial and temporal variability of storm activity^23–25^. The intensity of the storm that produced the preserved washover deposit is inferred from its landward extent, with more intense storms producing washover deposits that extend further landward from their coeval shoreline than less intense storms^23,25^. In addition, researchers have interpreted the stratigraphy of a washover deposit to provide information about the timing of deposition during the storm^15^ and the sediment source^26^. If washover deposits, particularly those that extend into back-barrier intertidal and subtidal areas, accrete significantly over time in response to multiple minor storm events, then the research community could be misinterpreting the meaning of some paleotempestites.

A large storm is typically thought of as the primary mechanism driving overwash; however, cross-island transport of water and sand is fundamentally a function of island geomorphology (height and width) and oceanographic conditions including tide, storm surge, wave setup, and wave runup^27^. Large storms are not a requirement for washover deposition in the backbarrier because with decreasing island width and elevation the resistance of a barrier to overwash decreases. Beach erosion, which impacts about 70% of Earth’s sandy beaches^28^, is the main driver of decreasing island width and elevation. Accelerating sea-level rise^29–31^, decreasing sediment supply^32,33^, anthropogenic influences^34^, and changing storm climate^35^ exacerbate beach erosion. This suggests that resistance to overwash should be decreasing globally. To better understand the transition of a barrier island from a coastal morphology that was resistant to overwash to one experiencing overwash and washover deposition, we present a 3-year time series of oceanographic conditions, overwash, and morphologic changes. The aim of this study is to test the assumption that washover fans and paleotempestites represent deposition during individual storm events by measuring the timescales over which washover accretion occurred at a barrier island site with an existing geologic record of overwash. Washover accretion is not necessarily driven by punctuated deposition during major storm events followed by longer-term recovery of island elevation and vegetated habitats. Alternatively, washover accretion could occur over timescales longer than an individual storm event caused by island morphology being conducive to frequent overwash.

## Methods

### Site description and previous work

**Figure 1:**
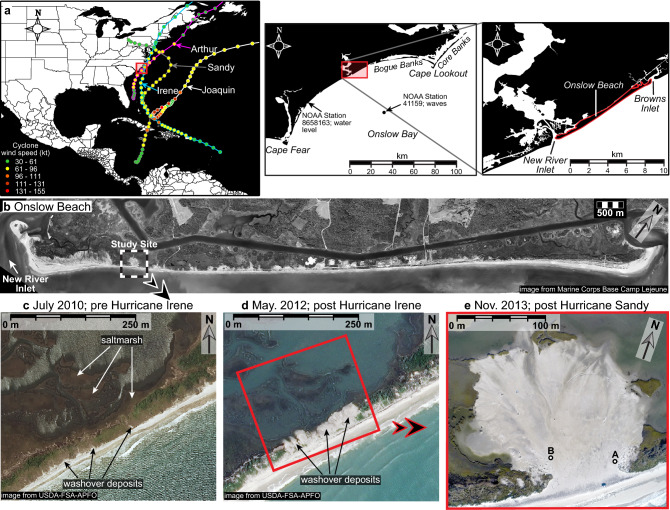
Locations of Onslow Beach and the study site. (**a**) The relevant hurricanes that impacted the site between August 2011 and November 2014 (maps created using Surfer^®^ 17.1.288; www.goldensoftware.com). (**b**) Image of Onslow Beach from April 2013 outlining the location of the site. (**c**) Numerous washover deposits were isolated to the coastal-dune area. (**d**) Hurricane Irene (August 27, 2011) caused washover deposits to extend landward and bury fringing saltmarsh. Photographs in (**c**,**d**) are from United States Department of Agriculture Farm Service Agency Aerial Photography Field Office (USDA-FSA-APFO). (**e**) Hurricane Sandy (October 29, 2012) caused overwash and formation of a large washover fan. Overwash-sensors located at A and B (photograph obtained using a drone).

The study site is located on Onslow Beach, NC (Fig. 1), which was part of a multi-year monitoring project of beach morphology that began in 2007. During that study, investigators mapped a narrow (supratidal cross-shore distance 45 m), low-elevation (max. 2.5 m relative to the North American Vertical Datum of 1988; NAVD 88) sector of the barrier that appeared to have a low resistance to overwash (same area as Site F2)^36^. Specifically, on May 5, 2011 the area was characterized by a 20-m wide beach from mean sea level to the scarped foredune toe, a 20-m wide foredune with a crest elevation of 2.5 m NAVD 88, and a back-dune area where elevations decreased linearly over a landward distance of 25 m to the saltmarsh at 0.4 m NAVD 88. The beach and dunes were eroding rapidly during the monitoring period, and based on linear regression, the mean sea level topographic contour on the beach moved landward an average of 5.96 m yr^−1^ during the period 2007–2012 (R^2^ value 0.69)^36^. The present monitoring study began after Hurricane Irene, which made landfall ~ 75 km north of Onslow Beach on August 27, 2011 as a Category 1 hurricane and caused overwash at Site F2 that buried back-barrier fringing saltmarsh (Fig. 1).

### Mapping the site

The site was mapped 16 times from May 21, 2012 to October 12, 2015 using a Riegl three-dimensional LMSZ210ii terrestrial laser scanner mounted on a truck^37^. The average time between mapping excursions was 83 days with a maximum and minimum of 175 days and 8 days, respectively (Supplementary Table S1). The scanner was set to emit around 2 million laser beams with about 1 million being reflected by objects and returned as x, y, and z data points per scan. Scan locations were positioned ~ 200 m apart. Data points were referenced using seven or nine surveyed and leveled reflectors (using a Trimble R8s GPS receiver) distributed around the area of each scan position. Field excursions were limited to the 2 h before and after low tide to maximize data coverage along the perimeter of the fan (the scanner cannot image through water).

Using Merrick Advanced Remote Sensing Software, we isolated ground points from the point clouds and created digital elevation models (DEMs) using Delaunay Triangulation. Those DEMs were imported into Golden Software’s Surfer with a 0.50-m grid spacing for analysis (Supplementary Figure S1). We consistently used the break in slope along the perimeter of the washover fan on each DEM to delineate it from adjacent lower-elevation saltmarsh and beach and higher-elevation dunes with an average digitizing error of 0.75 m. Washover area (W_a_) and volume (W_v_) were calculated from the DEMs. Error associated with measuring W_a_ (EW_a_) was defined as ± 1.25 × perimeter, with 1.25 being the sum of the DEM grid spacing and the digitizing error. We measured W_v_ using a DEM created from airborne lidar data collected in 2010^38^ as a constant basal surface. The 2010 DEM was subtracted from each successive DEM of the washover fan to calculate W_v_. The potential sources of error that could have impacted measuring W_v_ include GPS error, laser-scanner instrument error, error with manually levelling the reflectors and associating them with the surveyed points, error associated with editing the point cloud, and error associated with the interpolation algorithms used to create DEMs. We quantified these errors experimentally by scanning the same beach area three times during a 2-h period and creating DEMs (resulting vertical error = 0.043 m; see Supplementary Figure S2 for details). Measurement and procedural error associated with calculating W_v_ was defined as ± 0.043(W_a_ + EW_a_). Negative elevation change from compaction of the sediments must have occurred during the period, could not be quantified with our remote-sensing method, and is spatially heterogeneous, likely largest in landward areas where sand was deposited on top of saltmarsh peat. Volumes reported here should be considered minimum values because compaction was not addressed.

### Overwash processes

Overwash was recorded at two locations on the washover using HOBO water-level data loggers suspended in shallow wells (Fig. 1). The presence of overwash was validated visually with trail cameras programmed to take photographs every 5 min during daylight hours^22^. Overwash occurs when total water elevation exceeds the foredune ridge or beach berm elevation and is commonly parsed into a lower magnitude runup overwash regime, where wave runup overtops the dune or berm crest and an inundation overwash regime where the island is submerged^27,39^. Following the same methodology outlined in VanDusen et al.^22^, we recorded runup overwash, low-inundation overwash (water level < 10 cm above ground), and high inundation overwash (water level ≥ 10 cm above ground) from June 4, 2012 to July 16, 2015. The wells are located on the washover fan ~ 80 m apart in an along-beach direction and were initially installed at similar elevations (Fig. 1). As the monitoring progressed, the elevation of the ground around the wells fluctuated. The ground level 1.0 m away from Well A (period average = 0.91 ± 0.15 m; ± SD) generally increased through time resulting in that area becoming more resistant to overwash. The elevation of the ground around Well B (period average = 0.72 ± 0.07 m; ± SD) was generally lower than Well A. For this study we were interested in overwash that was most likely capable of transporting measurable volumes of sediment across the island and accurately classifying the overwash regime; therefore, we only included overwash events with a duration ≥ 30 min^22^. We created a composite overwash record such that if both wells experienced overwash, then we only included the highest water level and if one well recorded overwash, then we used that one well to characterize water level during the event. No data were recorded at Well B from October 24 to December 28, 2012 due to storm damage. Overwash at the site was placed in context with significant wave height (H_s_) and water-level data obtained from ndbc.noaa.gov, NOAA Station 41159 located 50 km southeast of the study area (buoy), and Wrightsville Pier NOAA Station 8658163 located about 55 km southwest of the study area (gauge; Fig. 1). Station 41159 was removed from service in 2015.

## Results

**Figure 2:**
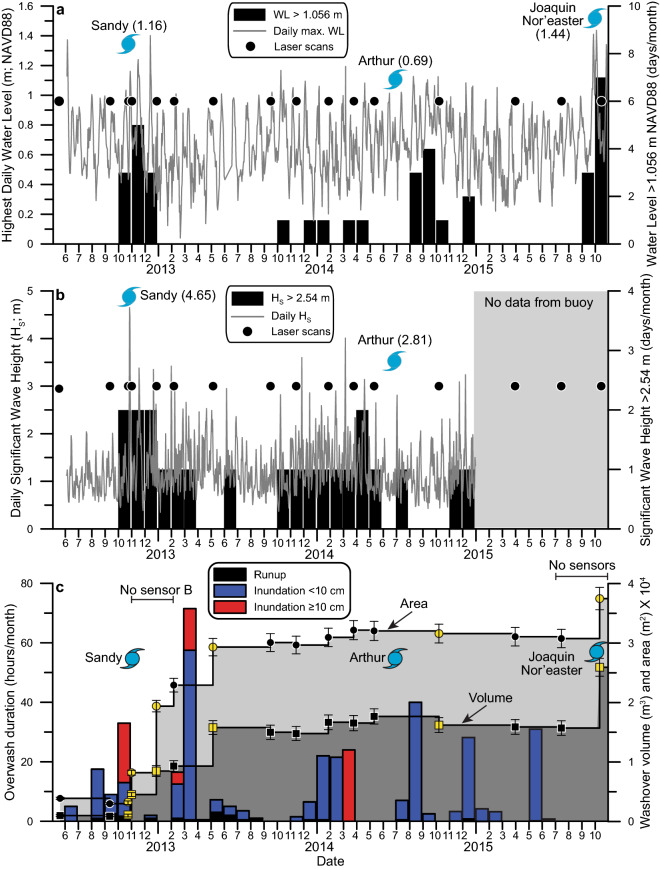
Time series of physical processes and data collection at the site from May 2012 to November 2014. (**a**) Highest daily water levels at NOAA station 8658163. (**b**) Daily significant wave heights at NOAA station 41159. (**c**) Overwash duration and water level at the site—a composite record from two sensors located on Fig. 1e. Measurements of area (circles) and volume (squares) were made using the DEMs and yellow points are measurements based on those DEMs shown in Fig. 3.

**Figure 3:**
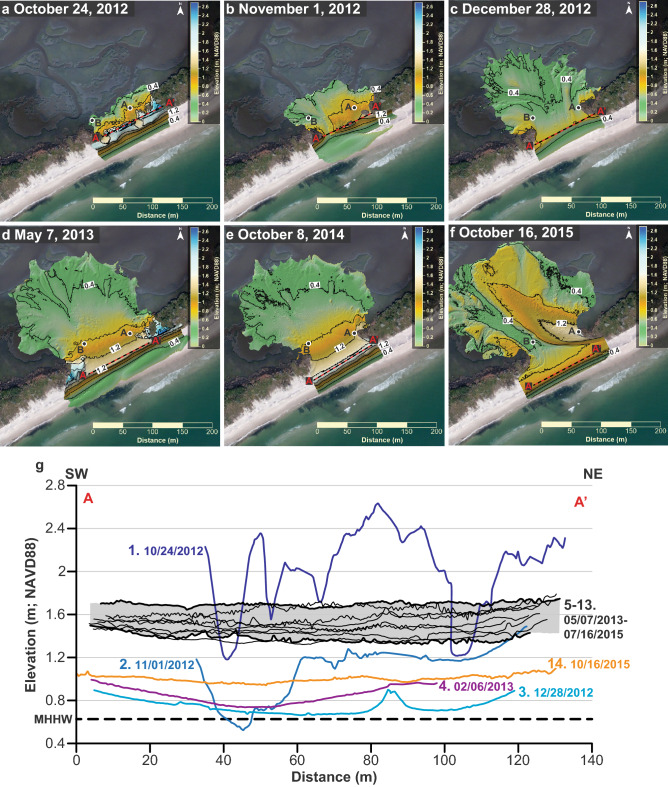
DEMs showing morphologic changes at the site. Background aerial photography from United States Department of Agriculture Farm Service Agency Aerial Photography Field Office (USDA-FSA-APFO) and maps were created using Surfer® 17.1.288 (www.goldensoftware.com). Circles show well locations. (**a**) Morphology of the site 5 days before Hurricane Sandy. (**b**) The washover deposit had more than doubled in size 2 days after Hurricane Sandy. (**c**,**d**) The washover deposit continued to grow during the subsequent 6 months. (**e**,**f**) The size of the washover deposit changed little until Hurricane Joaquin on October 12, 2015. (**g**) Along-shore elevation profiles extracted from all DEMs obtained. Profiles are from where the elevation is at a maximum (commonly the foredune crest) and numbered consecutively from October 2012. The gray shading highlights profiles from DEMs showing little change in washover area and volume (5–13; Supplementary Fig. S1). Dashed line = Mean Higher High Water (MHHW).

The site experienced multiple episodes of overwash, landward transport of sand, and washover fan lateral accretion during the 1240-day study period. Those episodes did not always occur simultaneously with a large storm. Initially, the study site experienced runup and low-inundation overwash during the months of June, August, September, and October of 2012 (Fig. 2). Overwash occurred through two throat channels that had cut through the foredune during previous storm events. From June through October 2012, overwash occurrence was neither related to stormy conditions nor increased the size of the washover fan (Figs. 2, 3). On October 24, 2012, the size of the washover fan was 3290 ± 165 m^2^ and 1116 ± 131 m^3^, using the 2010 DEM as a base, and the foredune was discontinuous with a maximum width of 11.5 m and an average height of 2.5 m (Figs. 2, 3). As Hurricane Sandy passed offshore of the area 5 days later on October 29, 2012, the maximum ocean water level and H_s_ was 1.16 m and 4.65 m, respectively, resulting in 20 h of high-inundation overwash that began in the afternoon of October 27, with ~ 20 cm of water measured above the ground surface at Well A. On November 1, 2 days after the storm passed, the washover fan had increased in size to 8204 ± 410 m^2^ and 4531 ± 327 m^3^ (Fig. 2). In comparison to the pre-storm survey, the foredune eroded, decreasing ~ 1.5 m in elevation, and the 0.04 m elevation contour of the beach moved landward a maximum and minimum of 25 m and 12 m, respectively (Fig. 3). Conditions associated with Hurricane Sandy deepened the southwestern throat channel near the location of Well B to an elevation below MHHW, inundated the area where the foredune previously existed, and transported sediment landward to produce the 149% and 306% increase in washover fan area and volume, respectively (Figs. 2, 3). Well B was damaged and not recording water levels from October 24 to December 28 because of high-velocity water flowing through the throat channel.

Over the next 2 months the site was impacted by two nor’easter events in the middle of November and the middle of December with a maximum ocean water level and H_s_ of 1.40 m and 3.35 m, respectively. During those 2 months, Well A recorded low-inundation overwash for 1 h on December 13 and runup overwash during peak high tides on December 14 and 15 (Fig. 2). Well B was inoperable at that time, but overwash was likely more frequent at that lower-elevation western end of the washover in the vicinity of the throat channel, than what was recorded by Well A. The washover fan grew during that 2-month period as it had during Hurricane Sandy. By December 28, the washover fan had increased in size to 19,396 ± 924 m^2^ and 8484 ± 873 m^3^. Accretion of the washover fan was likely due to overwash transporting sediment across the entire site because the strike-aligned profile sampled through the maximum elevations of the site decreased to a level close to MHHW at the transition between the foreshore and the landward-sloping washover (Figs. 2, 3). The 0.04 m elevation contour on the beach remained relatively stationary in comparison to the previous November 1 survey (Fig. 3).

The site experienced no overwash during the following month (January, 2013; Fig. 2) and the DEM of February 6, 2013 shows that the washover fan gained no volume (within error), but the beach had accreted (Fig. 3) and the 0.4 m contour moved seaward ~ 20 m to the pre-Hurricane Sandy position. After topography data were collected on February 6, 2013, the site experienced the highest frequency of overwash on record, with 4 days in February and 7 days in March for a total of 1.5 h of runup overwash, 68.5 h of low-inundation overwash, and 18 h of high-inundation overwash with a maximum water depth of 24 cm above ground level measured at Well B. During that 2-month period, no large storm waves or high-water events were recorded in the ocean (Fig. 2). The May 7, 2013 DEM shows that the washover fan increased in size to 29,321 ± 921 m^2^ and 15,790 ± 1300 m^3^ (Figs. 2, 3). Topography data for that May DEM was obtained 1 month after the overwash events occurred, and by that time a continuous narrow incipient foredune had established with an average elevation of 1.45 m (Fig. 3). During the 187 days after the post-Hurricane Sandy topography data were collected, the washover increased in area and volume 257% and 249%, respectively.

From May 7, 2013 to July 16, 2015 we mapped the topography of the site 8 times with a maximum and minimum period between scans of 175 and 47 days, respectively, and neither the area nor volume of the washover fan changed above the measurement error (Fig. 2). The strike-aligned profiles sampled through the maximum elevations of the site also showed little variation during that period, with average profile elevations ranging between 1.38 ± 0.04 and 1.70 ± 0.02 m NAVD 88 (Fig. 3g). Hurricane Arthur, a Category 2 storm, passed directly over the site in the middle of that period (July 4, 2014) but had little effect on the ocean waves, water level, or morphology of the site (Figs. 1, 2). The wells and water-level loggers were removed after the July 2015 topography survey because we thought ecological succession, foredune recovery, and backshore accretion would continue to increase resistance of the site to overwash; however, that was not the case. Hurricane Joaquin passed offshore of the site on October 4, 2015 as a Category 1 storm, coincided with a strong nor’easter, and produced an extended period of surge (Figs. 1, 2). Hurricane Joaquin reinitiated overwash of the site and expanded the size of the washover fan to 37,471 ± 1243 m^2^ and 25,927 ± 1664 m^3^ on October 12, 2015 (Figs. 2, 3). That was the last time we could access the island for field work, but aerial photography from other sources (e.g. the USGS and NOAA) showed that the site continued to overwash and the washover fan continued to expand landward and alongshore at least until August 2020.

## Discussion

The deposition of washover sediment during the study period at our site was not unprecedented. Although historical maps and aerial photography recorded no previous washover at the site since 1889, the geologic record shows that a single earlier washover deposit was preserved in the stratigraphy^40^. The landward portion of that earlier washover was sampled as a 40-cm thick sand bed in saltmarsh strata at a depth of 1.60 m, emplaced sometime between 1775 and 1807^40^. The presence of only one earlier washover suggests the site had been resistant to overwash capable of transporting sand across the dunes and into the back-barrier saltmarsh for ~ 200 years. The resistance of the site to overwash progressively decreased leading up to Hurricane Irene in 2011, a result of continuous beach erosion. The average rate of landward shoreline movement, based on linear regression, from 1875 to 2004 was 2.62 m year^−1^ (R^2^ = 0.93) and from November 2007 to May 2011 ~ 3880 m^3^ of sand was eroded from the foredune crest to the shoreline, defined as the 0.1 m NAVD 88 elevation contour^36^. Sea-level anomalies in 2009–2010 also facilitated erosion of the backshore and foreshore further decreasing the resistance of the site to overwash^41^. Eventually, with the beach and dunes narrowed, the maximum elevation of the site decreased to levels where overwash was imminent.

Washover deposition initiated at the study site during Hurricane Irene in 2011, but that was mainly the result of the morphology of the site being conducive to overwash as opposed to the Category 1 hurricane being an extraordinary event. A washover fan was deposited 500 m southwest of the study site in 1996 during Category 3 Hurricane Fran, which made landfall at Cape Fear 95 km southwest of Onslow Beach^40^. The beach and dunes of that southwestern Hurricane Fran washover area had accreted and built elevation by 2011 making that area resistant to overwash from Hurricane Irene. Similarly, the site examined in this study had recovered elevation since the storm event around 1790 and was resistant to overwash from Hurricane Fran. The impact of a storm on a barrier island commonly varies spatially and temporally due to along-shore variations in island morphology, the time scales over which an area accretes, the rate of shoreline movement, and the frequency and magnitude of erosive events^42–44^.

The washover fan examined here is not an event deposit, rather, it accreted throughout the 3-year study period and continues to accrete. Overwash transport of sediment was not only active during the largest storms, such as hurricanes, because of the site’s persistently low resistance to overwash. Hurricane Irene made landfall 60 km northeast of our site near Cape Lookout, and caused initial overwash and deposition of a small washover terrace at the site; however, most of the overwash and washover deposition happened after Hurricane Sandy, a large storm (Category 1), but one that passed 490 km offshore of the site and did not produce hurricane conditions locally. The washover deposit increased in area 257% and accreted landward 110 m during the 187 days after Hurricane Sandy, the result of frequent overwash during extra-tropical storms and spring tides. The occurrence of overwash in response to events other than major storms has been documented elsewhere, including along the eastern north Atlantic Coast^19^, the Pacific Coast of South America^20^ and the Gulf of Mexico Coast^45^, underscoring the capacity of overwash to transport sediment across a shoreline in the absence of local hurricane or tropical storm conditions. The adjacent Hurricane Fran washover area had a similar depositional history to the site examined here and after initial formation, the Hurricane Fran washover fan also increased in area and accreted laterally 120 m landward between 1998 and 2002, a period that included Hurricane Bonnie (1998) and multiple other tropical and extratropical storms^40^. Both washover deposits on Onslow Beach amalgamate numerous depositional events with overwash as the primary mechanism for transporting sediment as recorded in the center of the Hurricane Fran deposit as stacked fining-upward sand beds^40^. Composite washover deposits are not uncommon and have been recognized along other coastlines, including the coast of Denmark^46^, Australia^47,48^, and Louisiana, USA^49^; however, deposition of individual beds in those studies were attributed to large storm events as opposed to a low resistance of a shoreline to overwash. Typically, only one sand bed exists along the landward margin of composite washover deposits, the area where preservation potential is highest^14,40^. Deposition of that landward bed occurred during the overwash event that transported sand farthest landward; however, occurrence of overwash and landward accretion of a washover fan does not require tropical cyclone conditions.

The location, timing, and extent of washover deposition is controlled by site morphology, in addition to storm characteristics such as wind speed and storm track. The geological record can preserve washover deposits; however, interpreting what the wind, water-level, and wave conditions were like during deposition from mapping the extent of a paleo-washover sand bed or laminae could yield spurious results if the morphology of the island (width, height, beach slope) immediately preceding the storm is assumed to be uniform through time. The threshold elevation for overwash at the site varied from 0.52 to 1.64 m NAVD 88 during the monitoring period and was below 0.80 for 143 ± 45 days after October 24, 2012 when the site experienced the highest frequency of overwash and the washover fan increased the most in volume and area in the absence of large storms (Figs. 2, 3g). Furthermore, the threshold elevation for overwash was relatively high (1.32–1.64 m NAVD 88) during the period May 7, 2013–July 16, 2015 when the washover fan experienced little change in volume and area despite Hurricane Arthur passing directly over the site (Figs. 2, 3g). Changing beach morphology was an important factor that determined the occurrence of overwash and deposition of washover sediment at the site.

Many studies aimed at extending storm records into prehistorical time use a recent washover deposit and direct measurements of storm conditions during its deposition as a proxy for interpreting the geologic record, with the caveat that the geomorphology of the beach, dunes, and backbarrier are constant and recover rapidly between overwash events^22,23,50–52^. That assumption has received some criticism^53^. Accurate storm-impact assessments require beach slope and dune height to be constrained immediately preceding or during a storm, even when water level and wave characteristics are well constrained^54–56^. The difficulty in accurately predicting the modern occurrence of overwash without updated information on beach morphology suggests interpreting hurricane magnitude from a paleo-washover deposit using a modern washover as an analog could be misleading and increasingly so the further back in time a storm record extends. The assumption of uniform island morphology in paleo-storm records is difficult to circumvent because paleo-beach morphology can be impossible to reconstruct accurately. Confidence in the paleotempestologic method is provided by independently derived records from distinct locations along the Northwest Atlantic and Gulf of Mexico coasts that correspond well^25,57^. While assumptions are necessary in extending hurricane records beyond historical accounts and correspondence between the numerous records lends credence to the approach, paleotempestites alternatively could be indicating changes in storminess and/or a related decrease in the resistance of a shoreline to overwash at the multiyear time scale, as opposed to overwash associated with a specific magnitude or type of storm (hurricanes, tropical storms, nor’easters, etc.). The assumption that beach morphology is resilient and washover extent can be related to an individual storm is not applicable to Onslow Beach and likely other transgressive barrier islands.

## Conclusions

The Onslow Beach study area was resistant to overwash and washover deposition for 220 years prior to Hurricane Irene in 2011. During that period, beach and dune erosion continuously narrowed the site and decreased resistance to overwash until, around 2011 having crossed a morphologic threshold, overwash became a frequent occurrence. The volume and area of the washover fan increased rapidly, an average of 427 ± 28 m^3^ day^−1^ and 614 ± 28 m^2^ day^−1^ during the 8-day time step around Category 1 Hurricane Sandy, which passed far offshore of the site. Although the rate of washover fan accretion was highest during that short period around Hurricane Sandy, most of the volume and area gain occurred during the subsequent 187 days at lower average rates of 60 ± 2 m^3^ day^−1^ and 112 ± 5 m^2^ day^−1^. Most of the deposition of washover sediment occurred in the absence of large storms, mainly due to the low resistance of the site to overwash. Overwash and associated deposition of washover sediment is necessary for barrier island transgression but large storms are not a requirement. The impact of large storms on barrier islands is difficult to predict due to uncertainties in storm characteristics, and beach morphology at the time of influence, such as when Category 2 Hurricane Arthur passed directly over the site and caused no deposition of washover sediment. The areal extent and thickness of paleo washover fans preserved in the stratigraphic record is the product of both the resistance of a site to overwash (island morphology during storm impact) and the storm character (type and magnitude) that affected the site, thus caution should be exercised when interpreting these records in the context of individual major storm events. Furthermore, the time-series of overwash and washover extent presented here shows that large washover deposits can develop over a > 10-year period from multiple smaller overwash events and are not necessarily single major storm-event deposits.

## Supplementary information

Supplementary Information.

## Data Availability

The datasets generated during the current study are available in the Environmental Systems Science Data Infrastructure for a Virtual Ecosystem (ESS-DIVE) repository, https://data.ess-dive.lbl.gov/view/doi:10.15485/1660465 and are included in this published article (and its Supplementary Information files).
