# Supplementary material for: Long-term washover fan accretion on a transgressive barrier island challenges the assumption that paleotempestites represent individual tropical cyclones
Source: Sci Rep. 2020 Nov 12;10:19755. doi: 10.1038/s41598-020-76521-4 (PMC7661513; doi:10.1038/s41598-020-76521-4)
Supplement: Supplementary file 1 — Supplementary Information. [file 41598_2020_76521_MOESM1_ESM.pdf]

Supplementary Information for:

Long-term washover fan accretion on a transgressive barrier island challenges the assumption that paleotempestites represent individual tropical cyclones

Antonio B. Rodriguez[1*], Ethan J. Theuerkauf[2], Justin T. Ridge[3], Beth M. VanDusen[4], and Stephen R. Fegley[1]

[1]University of North Carolina at Chapel Hill, Institute of Marine Sciences, Morehead City, NC 28557, USA

[2]Michigan State University, Department of Geography, Environment, and Spatial Sciences East Lansing, MI 48824, USA

[3]Division of Marine Science and Conservation, Nicholas School of the Environment, Duke University Marine Laboratory, Beaufort, NC 28516, USA

[4]University of Michigan, School for Environment and Sustainability, Ann Arbor, MI 48109, USA

*Corresponding author- email: abrodrig@email.unc.edu, Phone: 252-726-6841

Supplementary Table 1: Washover fan size through time using a 2010 digital elevation model as a constant basal surface.

Date	Area (m^2)	Volume (m^3)
5/21/2012	3890 ±370	998 ±183
9/12/2012	2997 ±320	885 ±143
10/24/2012	3290 ±393	1116 ±158
11/1/2012	8204 ±618	4531 ±379
12/28/2012	19396 ±924	8484 ±874
2/6/2013	22922 ±828	9314 ±1021
5/7/2013	29321 ±921	15790 ±1300
9/16/2013	30082 ±950	15004 ±1334
11/14/2013	29668 ±1024	14788 ±1320
1/27/2014	30949 ±1035	16678 ±1375
3/26/2014	32173 ±1048	16548 ±1428
5/12/2014	32039 ±1104	17665 ±1425
10/8/2014	31595 ±1073	16208 ±1405
4/1/2015	31065 ±1086	15896 ±1383
7/16/2015	30761 ±1035	15713 ±1367
10/12/2015	37471 ±1244	25927 ±1665

Supplementary Figure 1: Digital elevation models of the site through time. Background aerial photography from United States Department of Agriculture Farm Service Agency Aerial Photography Field Office (USDA-FSA-APFO) and maps were created using Surfer ® 17.1.288 (www.goldensoftware.com).

May 21, 2012

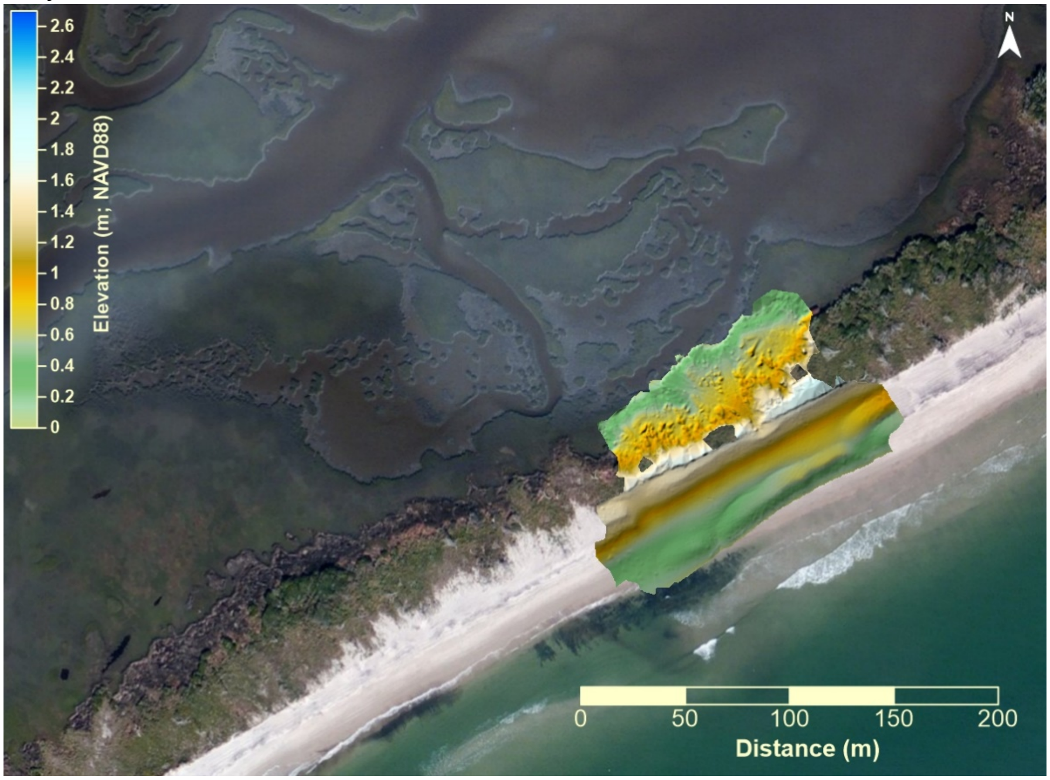

September 12, 2012

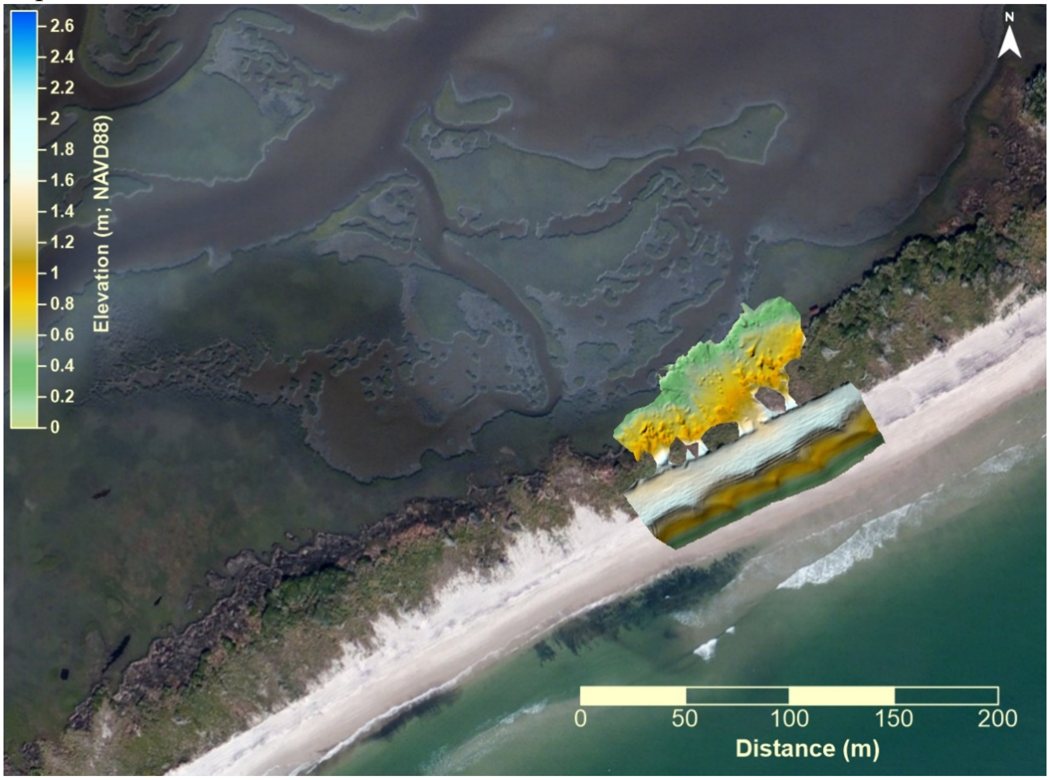

October 24, 2012

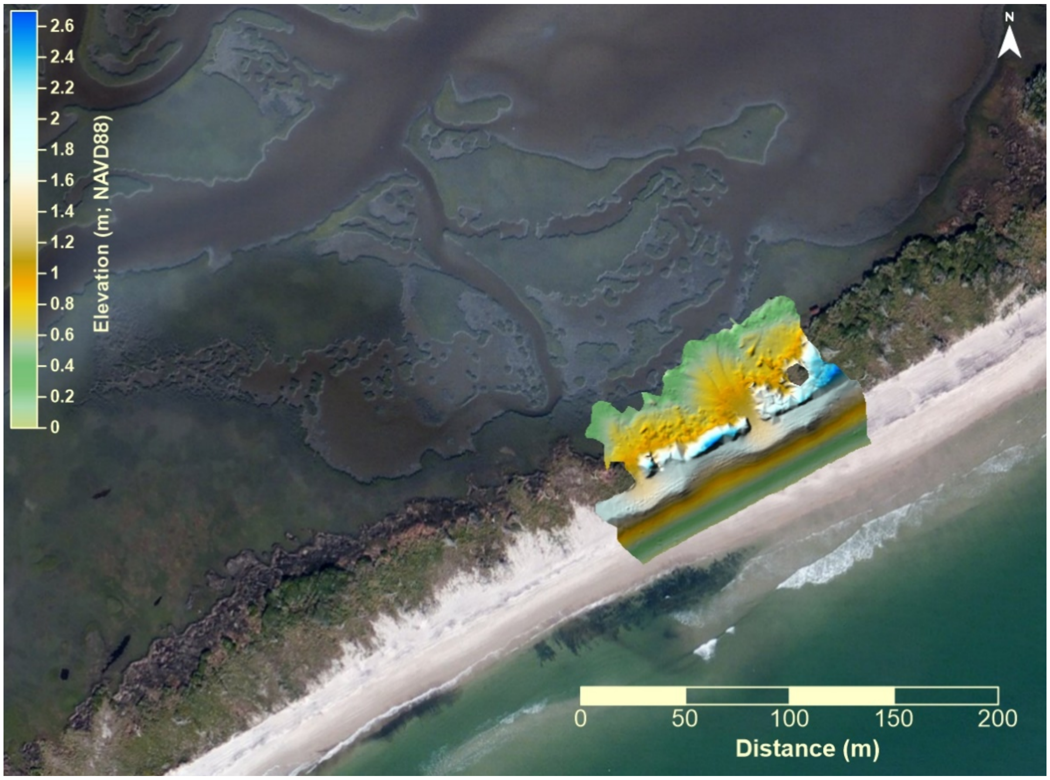

November 1, 2012

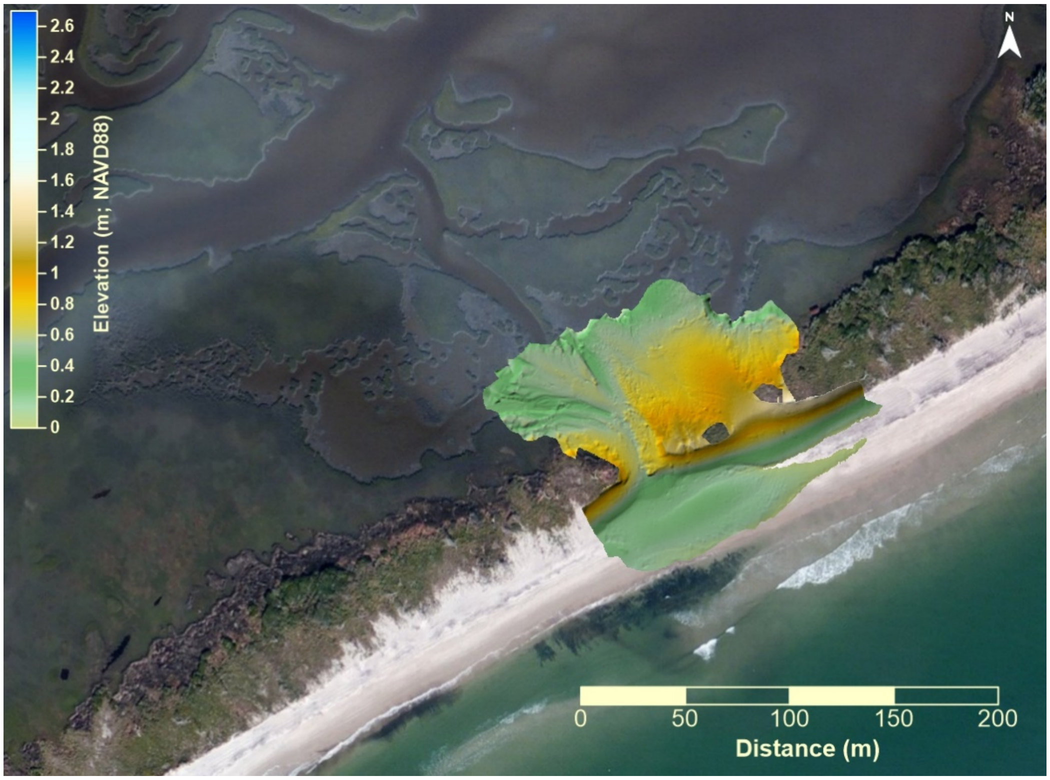

December 28, 2012

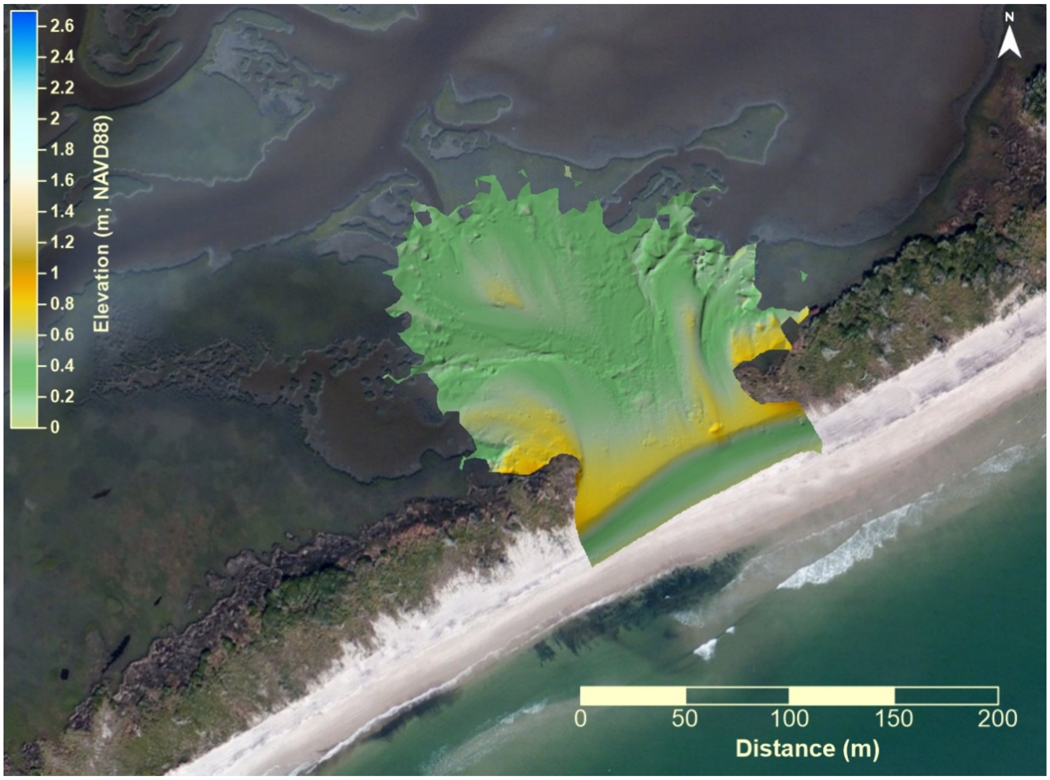

February 6, 2013

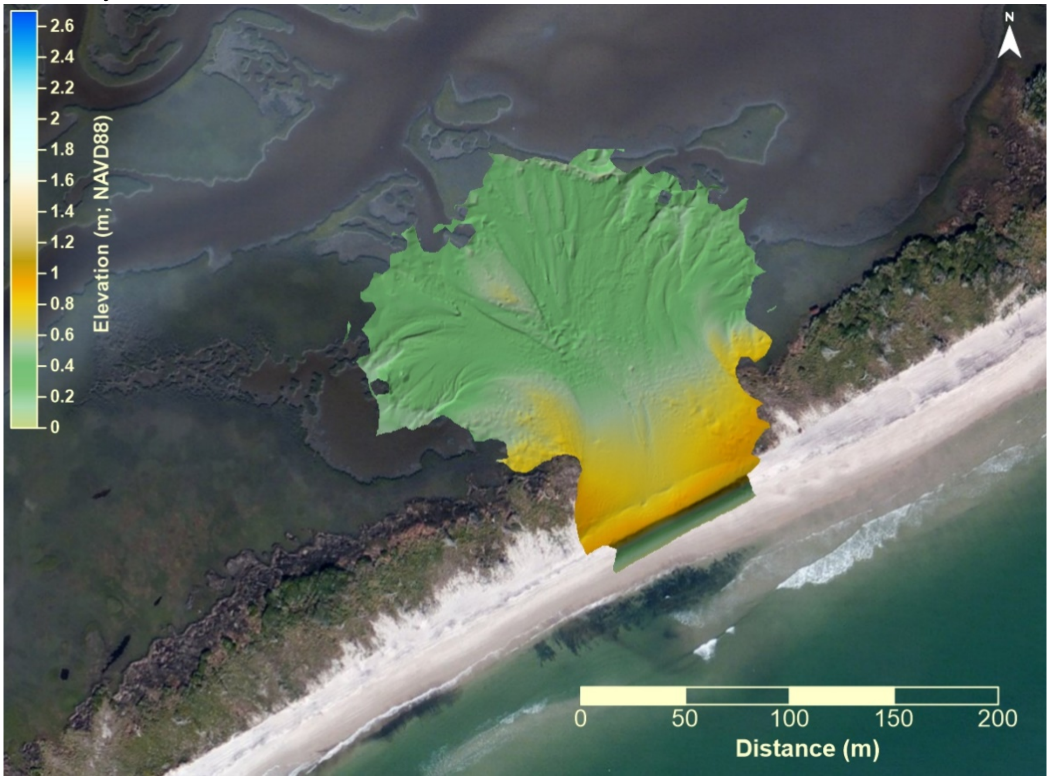

May 7, 2013

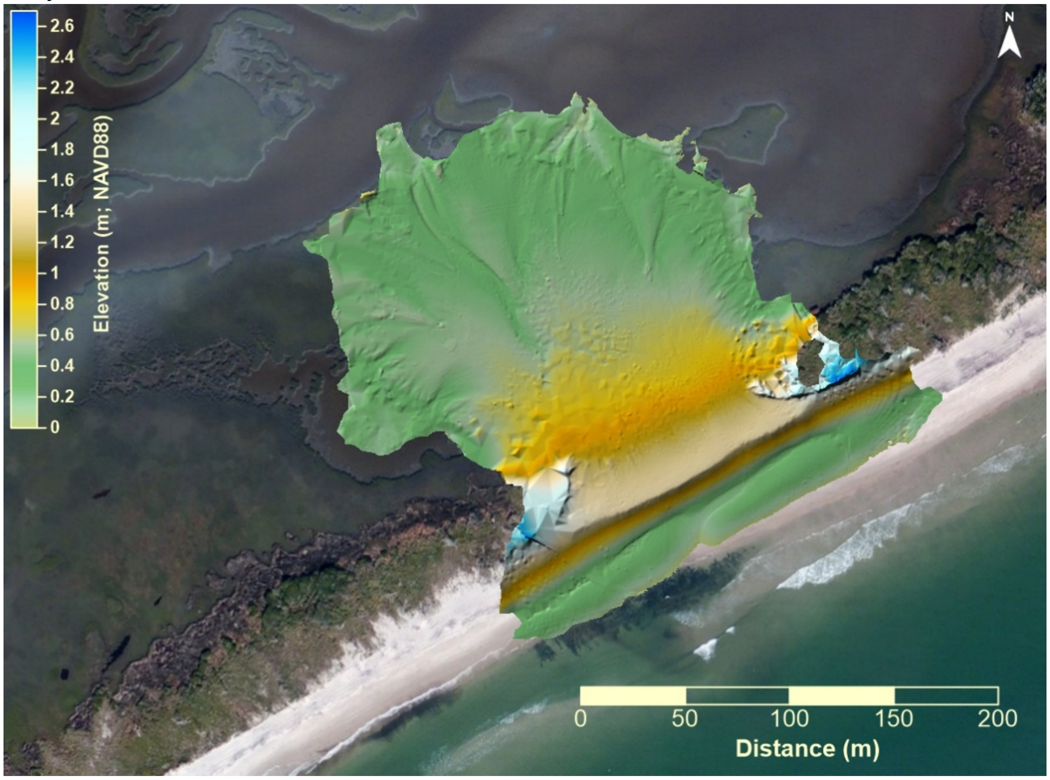

September 16, 2013

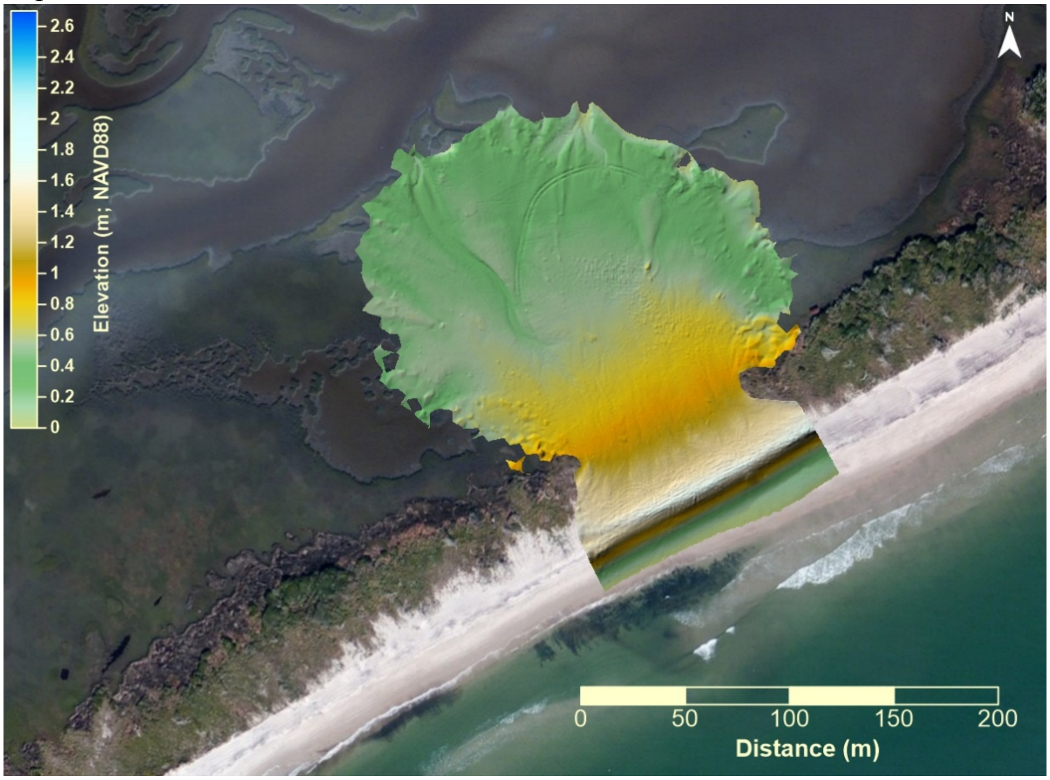

November 14, 2013

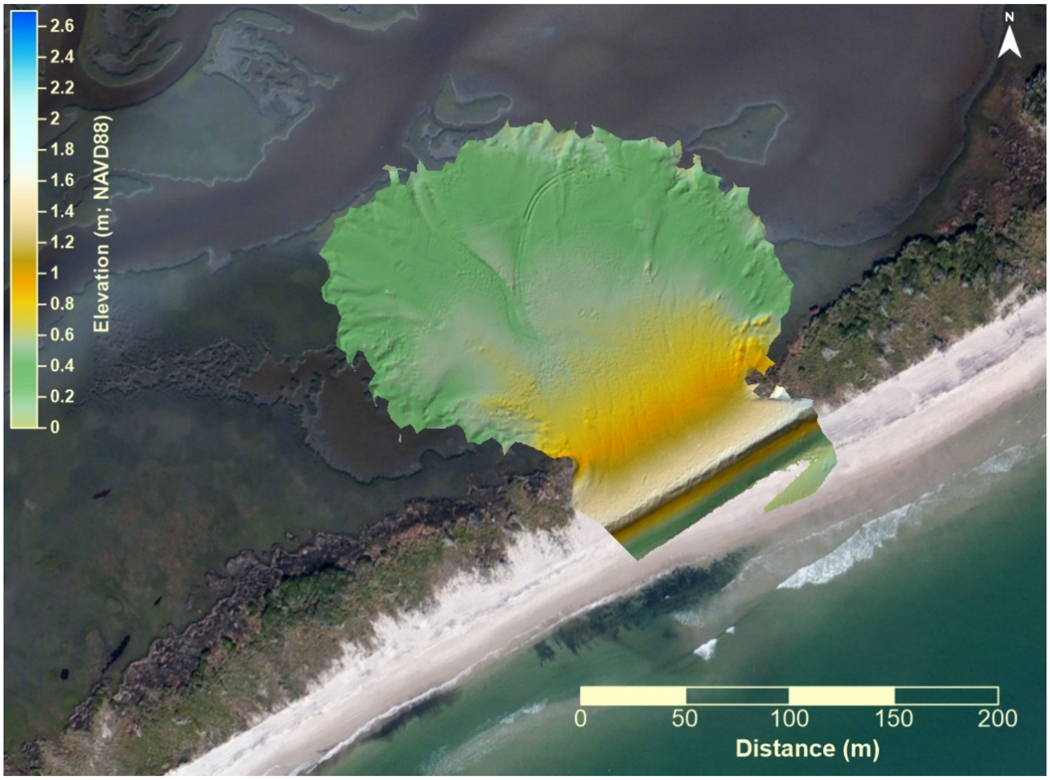

January 27, 2014

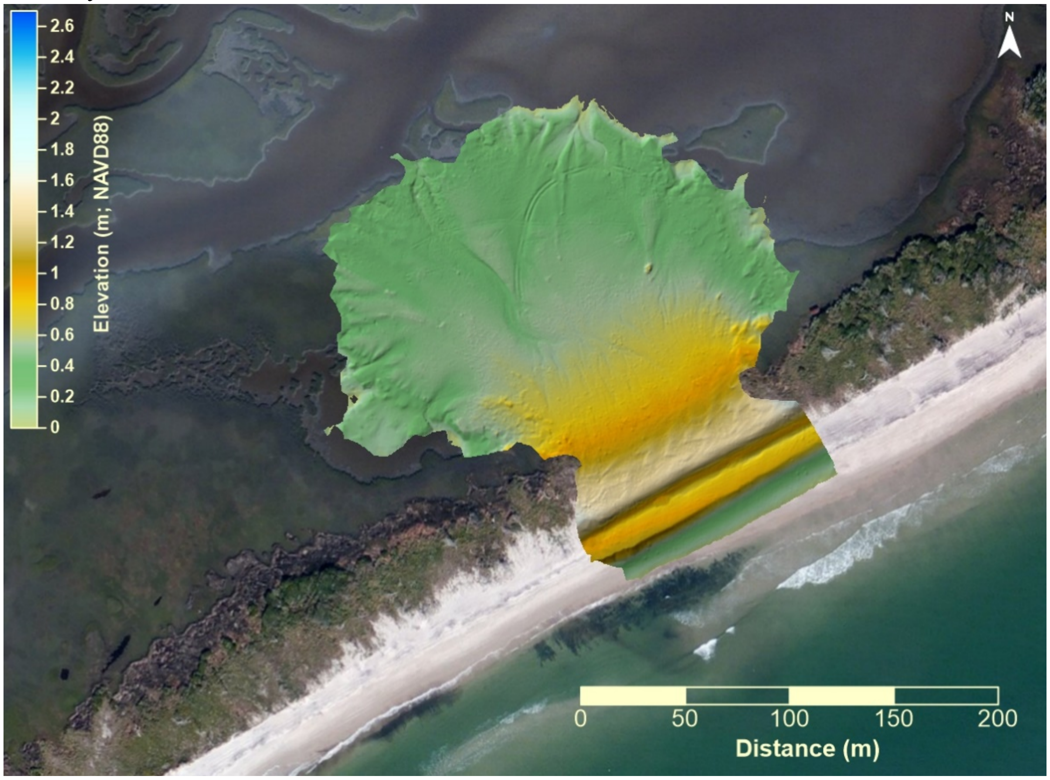

March 26, 2014

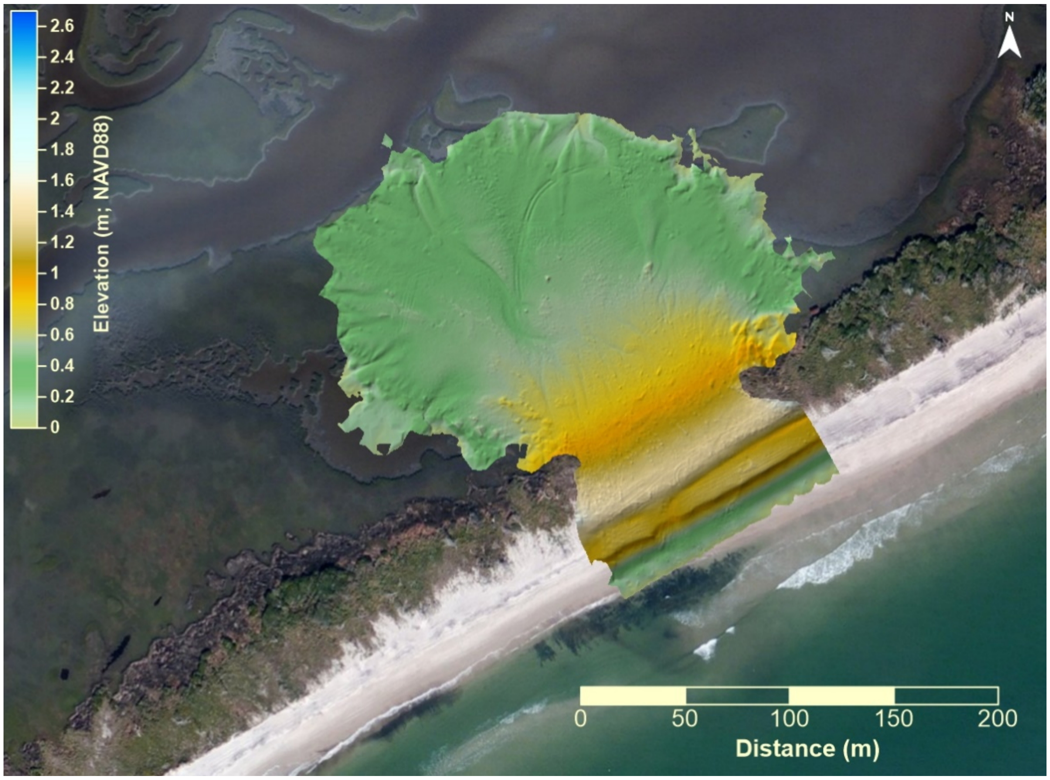

May 12, 2014

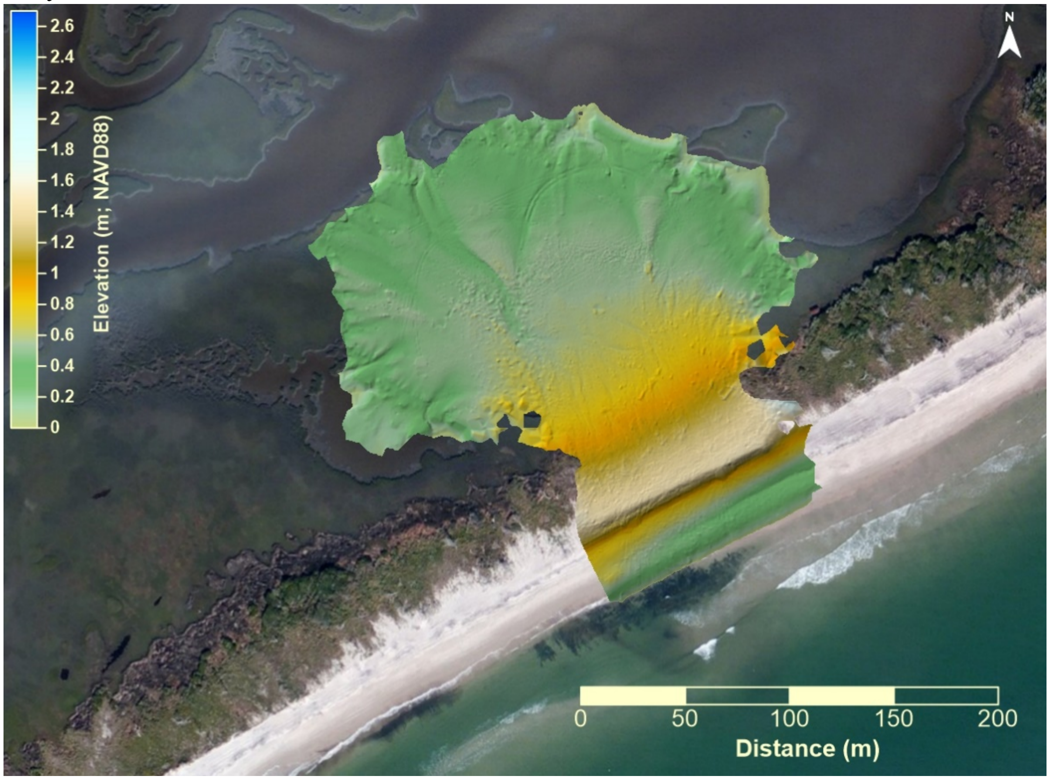

October 8, 2014

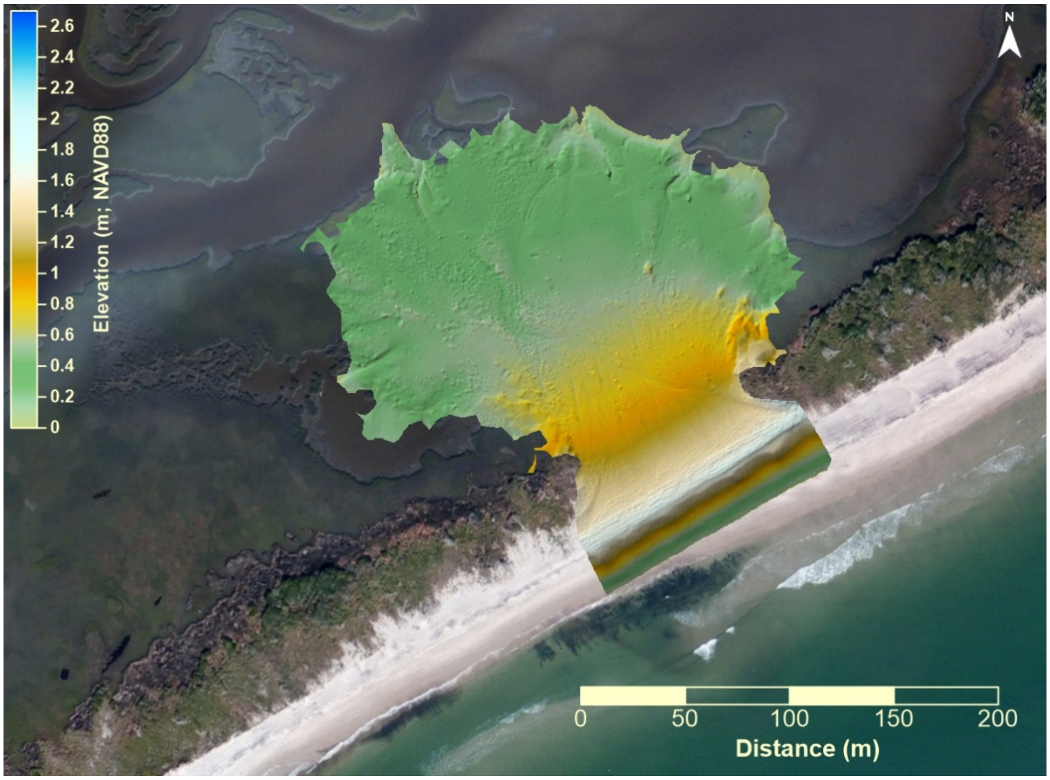

April 1, 2015

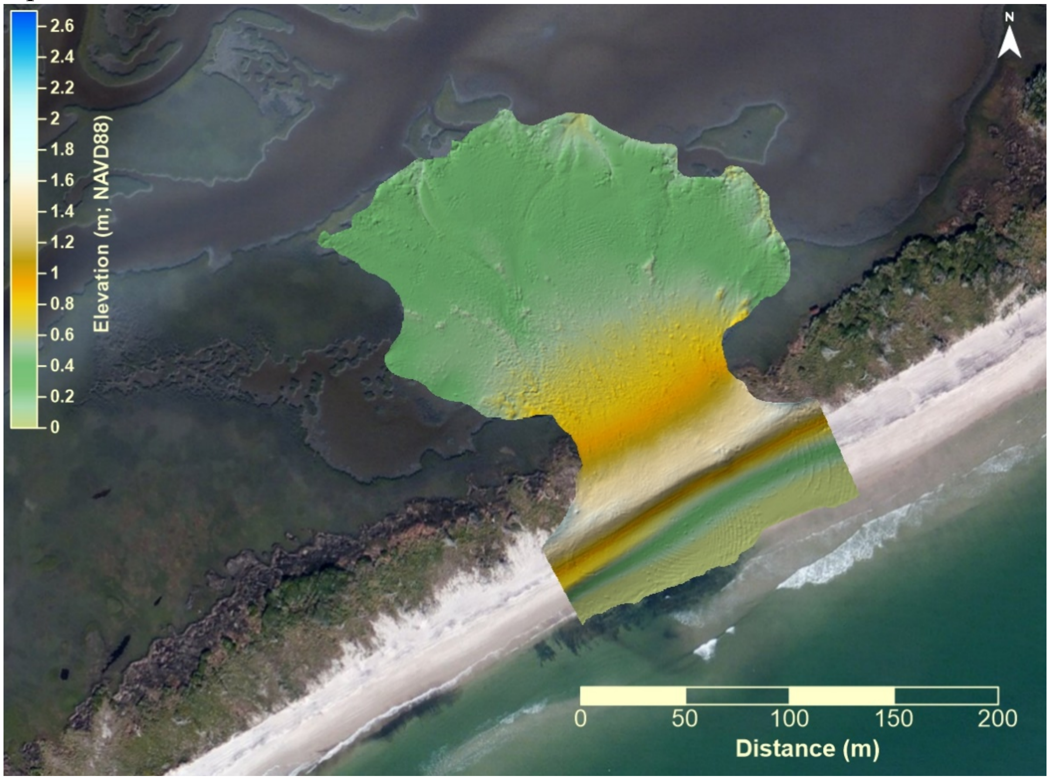

July 16, 2015

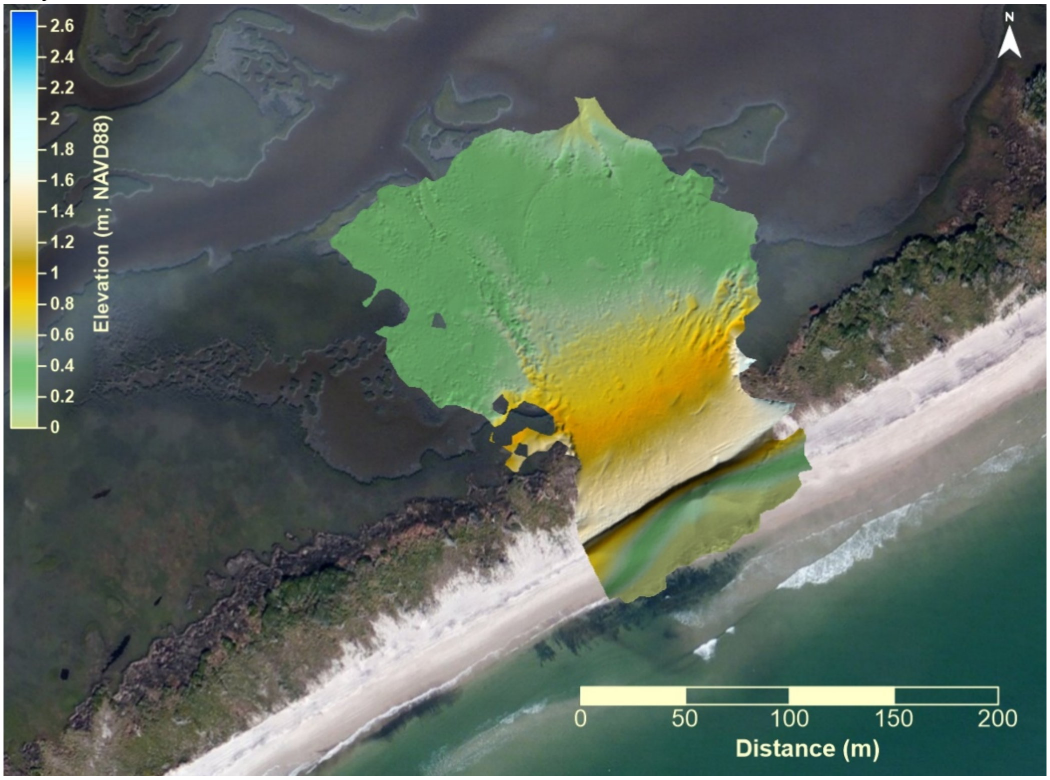

October 12, 2015

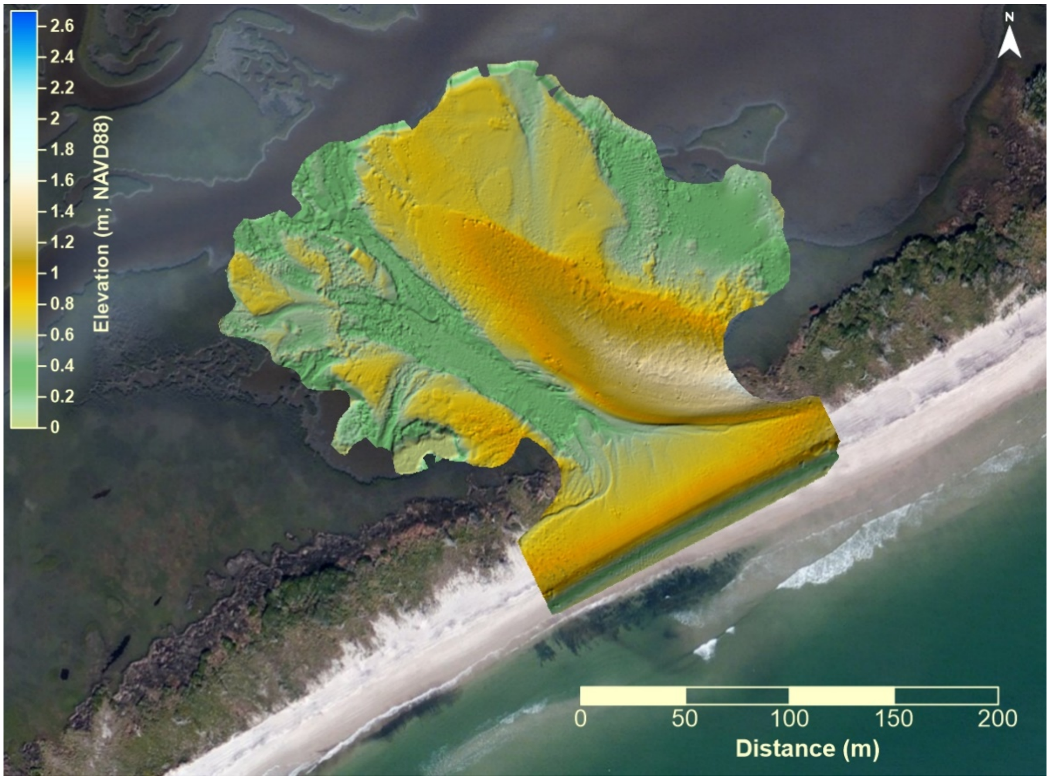

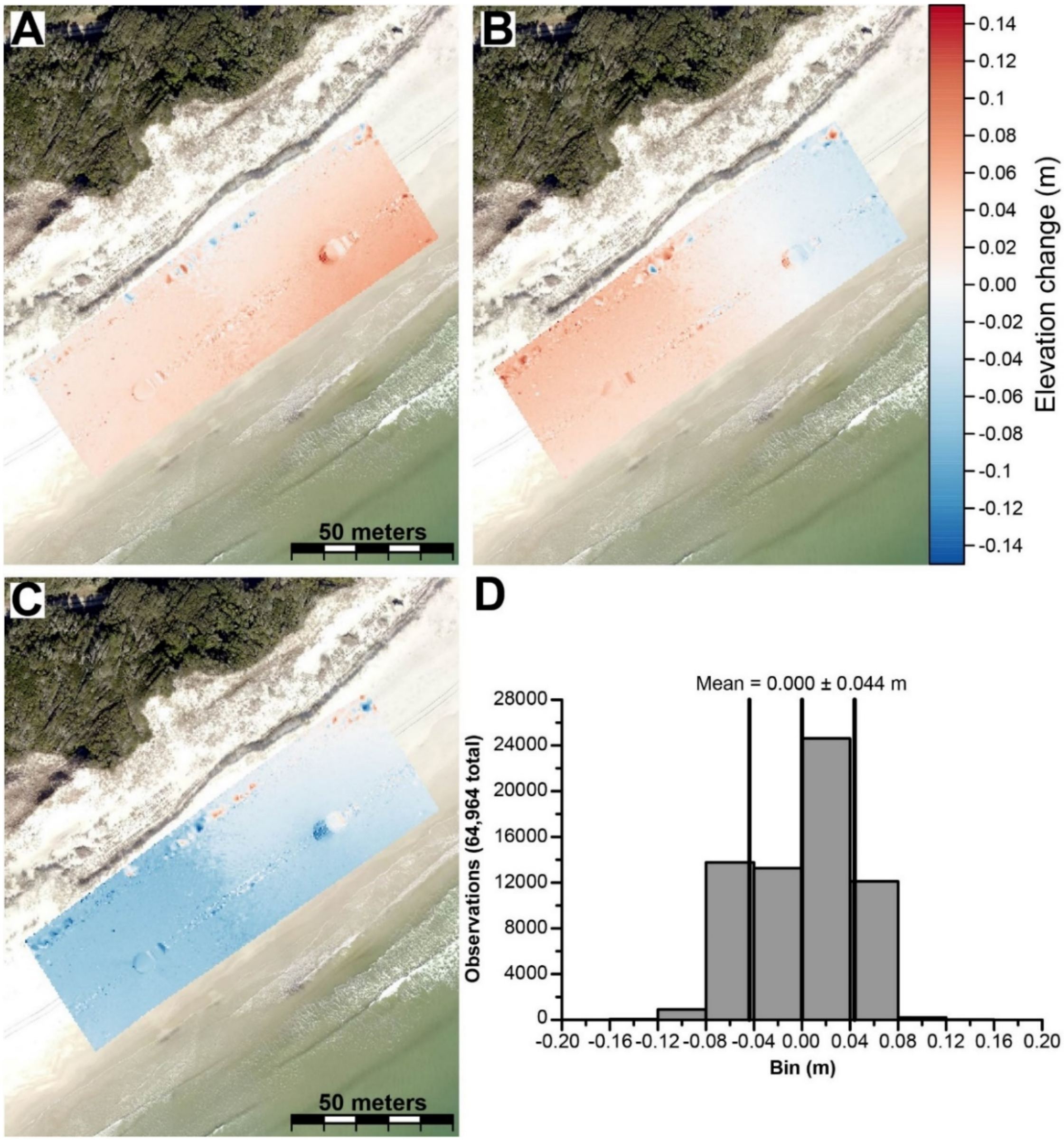

Supplementary Figure 2: We quantified GPS error, laser-scanner instrument error, error with manually levelling the reflectors and associating them with the surveyed points, error associated with editing the point cloud, and error associated with the interpolation algorithms used to create DEMs experimentally by scanning and creating DEMs of the same beach area three times. Each set-up included two scan positions and repositioning and resurveying 8 reflectors. Those three datasets were processed identically, using the same methods outlined above and in the paper, to create DEMs and vertical error was estimated by subtracting grid cells (A-C). The mean of the 64,959 grid-cell values of the subtractions was ~zero indicating no bias exists (D). Vertical error was defined as 0.043 m, the mean standard deviation of the elevation differences between grid cells. Background aerial photography from United States Department of Agriculture Farm Service Agency Aerial Photography Field Office (USDA-FSA-APFO) and maps were created using Surfer ® 17.1.288 (www.goldensoftware.com).
